# JAK2 as a surface marker for enrichment of human pluripotent stem cells-derived ventricular cardiomyocytes

**DOI:** 10.1186/s13287-023-03610-2

**Published:** 2023-12-13

**Authors:** Lee Chuen Liew, Boon Min Poh, Omer An, Beatrice Xuan Ho, Christina Ying Yan Lim, Jeremy Kah Sheng Pang, Leslie Y. Beh, Henry He Yang, Boon-Seng Soh

**Affiliations:** 1 Institute of Molecular and Cell Biology (IMCB), Agency for Science, Technology and Research (A*STAR), 61 Biopolis Drive, Proteos, Singapore, 138673 Republic of Singapore; 2 Cancer Science Institute of Singapore, National University of Singapore, Singapore, 117599 Republic of Singapore; 3 Department of Biological Sciences, National University of Singapore, Singapore, 117543 Republic of Singapore

**Keywords:** Cardiac differentiation, Human pluripotent stem cells, Ventricular cardiomyocytes, Cell surface marker, Cardiac subtypes, JAK2

## Abstract

**Background:**

Human pluripotent stem cell (hPSC)-derived cardiomyocytes (CMs) hold great promise for cardiac disease modelling, drug discovery and regenerative medicine. Despite the advancement in various differentiation protocols, the heterogeneity of the generated population composed of diverse cardiac subtypes poses a significant challenge to their practical applications. Mixed populations of cardiac subtypes can compromise disease modelling and drug discovery, while transplanting them may lead to undesired arrhythmias as they may not integrate and synchronize with the host tissue's contractility. It is therefore crucial to identify cell surface markers that could enable high purity of ventricular CMs for subsequent applications.

**Methods:**

By exploiting the fact that immature CMs expressing myosin light chain 2A (MLC2A) will gradually express myosin light chain 2 V (MLC2V) protein as they mature towards ventricular fate, we isolated signal regulatory protein alpha (SIRPA)-positive CMs expressing intracellular MLC2A or MLC2V using MARIS (method for analysing RNA following intracellular sorting). Subsequently, RNA sequencing analysis was performed to examine the gene expression profile of MLC2A + and MLC2V + sorted CMs. We identified genes that were significantly up-regulated in MLC2V + samples to be potential surface marker candidates for ventricular specification. To validate these surface markers, we performed immunostaining and western blot analysis to measure MLC2A and MLC2V protein expressions in SIRPA + CMs that were either positive or negative for the putative surface markers, JAK2 (Janus kinase 2) or CD200. We then characterized the electrophysiological properties of surface marker-sorted CMs, using fluo-4 AM, a green-fluorescent calcium indicator, to measure the cellular calcium transient at the single cell level. For functional validation, we investigated the response of the surface marker-sorted CMs to vernakalant, an atrial-selective anti-arrhythmic agent.

**Results:**

In this study, while JAK2 and CD200 were identified as potential surface markers for the purification of ventricular-like CMs, the SIRPA+/JAK2+ population showed a higher percentage of MLC2V-expressing cells (~ 90%) compared to SIRPA+/CD200+ population (~ 75%). SIRPA+/JAK2+ sorted CMs exhibited ventricular-like electrophysiological properties, including slower beating rate, slower calcium depolarization and longer calcium repolarization duration. Importantly, vernakalant had limited to no significant effect on the calcium repolarization duration of SIRPA+/JAK2+ population, indicating their enrichment for ventricular-like CMs.

**Conclusion:**

Our study lays the groundwork for the identification of cardiac subtype surface markers that allow purification of cardiomyocyte sub-populations. Our findings suggest that JAK2 can be employed as a cell surface marker for enrichment of hPSC-derived ventricular-like CMs.

**Supplementary Information:**

The online version contains supplementary material available at 10.1186/s13287-023-03610-2.

## Introduction

The discovery of human pluripotent stem cells, including human embryonic stem cells (hESCs) and human-induced pluripotent stem cells (hiPSCs) [1, 2], has opened a new avenue for biomedical research, ranging from cell therapy to disease modelling, and drug screening, owing to their plasticity to be able to give rise to almost all of the clinically relevant cell types. Cardiovascular diseases (CVDs), being one of the leading causes of death worldwide, are among the diseases that are extensively studied using hPSCs. Particularly, the generation of many CVD models, both in vitro and in vivo, has provided useful molecular insights to heart diseases [3–5]. However, hPSCs-derived cardiomyocytes (hPSC-CMs) generated from the current differentiation protocols remain highly heterogeneous, typically giving rise to different cardiac subtypes such as atrial-, ventricular- and pacemaker-like cells [6, 7]. These different subtypes of cardiomyocytes that exhibited different phenotypes, electrophysiological properties and functions [7–9] represent the major challenge for downstream applications of hPSC-CMs. For instance, the use of mixed population of cardiomyocytes in modelling chamber-specific cardiac disease may confound the disease phenotypes and consequently compromise the credibility of the drug response outcome [4], which in turn leads to limited success in drug discovery for region-specific heart diseases. More importantly, transplanting a heterogeneous pool of hPSC-CMs into an infarcted heart might disturb normal electrical impulse propagation in the heart, leading to undesirable therapeutic outcomes, such as arrhythmias [10]. Therefore, the prerequisite for translational applications of hPSC-CMs is to generate and isolate a relatively pure population of cardiomyocyte subtype for subsequent applications.

Over the last few decades, there have been numerous reports on advancements in differentiating cardiomyocyte subtypes. For instance, retinoic acid (RA) has been identified as a crucial factor in specifying atrial cell fate [11, 12], while inhibition of the canonical Wnt pathway with IWR-1 has been shown to promote differentiation towards ventricular cardiomyocytes [13]. Nonetheless, the challenge of obtaining a homogeneous population of cells still persists, as differentiation protocols appear to be cell line-dependent. On top of modulating relevant signalling pathways via small molecules, hPSC line harbouring fluorescent reporter driven by the transcriptional control of human MYL2 promoter (MLC2V) [14, 15] and chick ovalbumin upstream promoter transcription factor II (COUP-TFII) [16] were also employed to facilitate the isolation of ventricular and atrial cardiomyocytes, respectively. However, the major drawback of such cardiomyocytes is the involvement of virus-mediated gene transfer that has raised safety issues such as unknown off-target mutagenesis or immunogenicity and therefore holding them back from further clinical translation.

To overcome the challenge of cellular heterogeneity without genetic manipulation, we sought to identify novel cell surface markers for live cell sorting of ventricular-specific CMs. Through RNA isolation of SIRPA + CMs that were sorted based on intracellular MLC2A or MLC2V expression using MARIS, we performed RNA sequencing and identified JAK2 as a potential surface marker for purification of ventricular-like cardiomyocytes. We showed that SIRPA+/JAK2+ cell population was enriched for ventricular-like CMs, in both H7 and BJ lines (~ 90%). These SIRPA+/JAK2+ sorted CMs expressed high level of ventricular-specific marker, MLC2V, validated through immunofluorescent staining and western blot analysis. Additionally, electrophysiological characterization of the SIRPA+/JAK2+ sorted CMs demonstrated properties similar to ventricular CMs, such as prolonged calcium repolarization phase. Importantly, SIRPA+/JAK2+ sorted cardiomyocytes, which are ventricular-like CM, did not respond to vernakalant treatment, an atrial-selective anti-arrhythmic agent. Therefore, our findings suggest that JAK2 can be employed as a cell-surface marker to facilitate the isolation of hPSC-derived ventricular cardiomyocytes from the heterogeneous CMs cultures.

## Methods

### Pluripotent stem cell lines culture

The human ESC lines, H7 and ES03, and human iPSC line derived from BJ fibroblasts (all cell lines were purchased from WiCell) were cultured on Matrigel (Corning, USA)-coated plates with StemMACS™ iPS-Brew XF medium (Miltenyi Biotec). Culture medium was changed daily, and cells were passaged using ReLeSR (STEMCELL Technologies) after reaching 80–90% confluency.

### Cardiomyocyte differentiation, selection and maturation

For cardiomyocyte differentiation, pluripotent stem cells were dissociated into single cells using Accutase (Nacalai-Tesque) and seeded on Matrigel-coated plates in StemMACS™ iPS-Brew XF. At 90–95% confluency, cardiac differentiation was induced by modulating Wnt signalling using Gsk-3 (glycogen synthase kinase 3) and Wnt inhibitors, as described by Lian et al., 2013 [17]. At Day 7, the media was replaced with RPMI1640 containing MACS NeuroBrew-21 (Miltenyi Biotec) and refreshed every 2 days. At day 16, PSC-CMs were then cultured in glucose-depleted culture medium supplemented with fatty acid (GFAM) for cardiomyocyte selection and metabolic maturation. The GFAM medium contains RPMI without glucose supplemented with MACS NeuroBrew-21, galactose (10 mM), oleic acid (100 µM) and palmitate acid (50 µM) [18].

### Isolation of MLC2A + and MLC2V + CMs by MARIS

MLC2A + and MLC2V + CMs were isolated from PSC-CMs using a modified MARIS method, as previously reported [19, 20]. Briefly, at 3 weeks post-contraction, hPSC-CMs were dissociated into single cells using Accutase. Cells were then fixed and permed with 4% paraformaldehyde (PFA). Anti-human CD172a/b (SIRPα/β)-PECy7 (1:200; 323,808; Biolegend), anti-MLC2a-FITC (1:10; 130–106-141; Miltenyi Biotec) and anti-MLC2v-PE (1:10; 130–106-133; Miltenyi Biotec) antibodies were diluted in blocking buffer (1% bovine serum albumin (BSA)) and 0.2% saponin (Sigma) in phosphate-buffered saline (PBS) supplemented with 50 U/ml Superase. In (Ambion) and incubated with the cell suspension for 1 h. The cells were then washed with Dulbecco’s PBS (DPBS) before FACS on BD FACS Aria. Sorted cells were treated with Proteinase K for 1 h at 50 °C before addition of Trizol LS (Invitrogen) for RNA extraction. DNase treatment using Turbo DNA-free (Ambion) was performed to remove contaminating genomic DNA.

### RNA sequencing

#### Identification of ventricular-specific surface marker

Gene expression profiling has been performed by using CSI NGS Portal [21]. Briefly, raw fastq files were trimmed by Trimmomatic [22] for adapter removal. The clean reads were aligned to the reference human genome (hg19) by using STAR [23] (v2.7.3a) with default parameters. The gene expression quantification was done by using HTSeq-count [24] (v0.11.2) in strand-specific mode “-s reverse” to obtain raw read counts for each gene, and read counts only from the sense strand are used. First, hierarchical clustering was performed with regionReport [25] to compare the gene expression profiles of the samples, by using the top 500 genes with the highest variance across the samples. Then, the differential gene expression analysis was performed by using DESeq2 [26] (v1.24.0) starting from the raw read counts by comparing BJ MLC2V samples to BJ MLC2A samples after collapsing the samples within each group (2 for BJ MLC2V and 3 for BJ MLC2A samples) as replicates. The genes that are not expressed or lowly expressed (read counts <  = 2 on average per sample) were removed from the analysis. In total, 1349 and 1516 genes were significantly up- and down-regulated, respectively, in the BJ MLC2V samples compared to BJ MLC2A samples, after correction for multiple hypothesis testing (log2 fold change > 1, P_adj_ < 0.05, using Benjamini–Hochberg method). The same analysis was repeated to for ESO3 MLC2V and BJ MLC2A samples; 1904 and 2811 genes were significantly up- and down-regulated, respectively. Genes that were up-regulated in both sets of comparisons, and were found to encode for surface markers were identified as potential surface marker candidates for ventricular specification. The top 30 genes were then used for heatmap plotting in R.

#### Gene expression profiling of SIRPA+/JAK2+ sorted CMs, MYL2-TdTomato reporter-derived ventricular CMs, adult human ventricle and atrial samples

RNA extraction from SIRPA+/JAK2+ and MYL2-TdTomato + sorted CMs was performed using Trizol (Invitrogen), followed by DNase treatment with Turbo DNA-free (Ambion) to eliminate contaminating genomic DNA. RNA-seq data from adult human atrial and ventricular samples were obtained from an existing database (GEO accession: GSE81585). These RNA-seq data were then quality trimmed and filtered using fastp [27, 28] with default settings. RNA-seq reads were then mapped to the GRCh38 reference human genome assembly using STAR [23] with default parameters, using GENCODE v38 annotations. FPKM values were then tabulated using FPKM Count in the RSeQC package [29] and averaged across biological replicates. Raw counts were processed following the vignette of the DESeq2 package [30]. Briefly, raw counts were read using the DESeq2 package and pre-filtered to remove genes that have less than 10 counts across all samples. The human atria and ventricle samples were contrasted to identify the top 100 up-regulated genes in the atria and ventricle, with an adjusted p value < 0.05 and a log2 fold change > 1. Variance stabilizing transformation was used to stabilize the variance across the means of all samples. Gene expression heatmaps were plotted using normalized and scaled gene expression levels across all samples.

### Florescence activated cell sorting (FACS)

Cardiomyocyte (at least day 45 of culture) were dissociated using Accutase. Cells were then co-stained with anti-human CD172a/b (SIRPα/β)-conjugated antibody (1:200; 323,808; Biolegend), and either anti-human CD200 FITC-conjugated antibody (1:20; 130–106-015; Miltenyi Biotec), or rabbit-anti-JAK2 unconjugated antibody (1:20; PA511267; Invitrogen) in blocking buffer (1% foetal bovine serum (FBS) in PBS) for 1 h at 37 °C. The secondary antibody used was Alexa Fluor 488-conjugated donkey anti-rabbit (1:1000; Thermo Scientific). Stained cardiomyocytes were washed thoroughly with PBS before the resuspended in FACs buffer (0.5% FBS and 1% BSA in PBS). FACS was performed using BD FACSAria II or BD FACSAria™ Fusion Flow Cytometer (BD Bioscience). Unstained sample was used as control populations.

### Immunostaining

Surface marker-sorted cardiomyocytes were seeded on a 96-well plate coated with Matrigel at a density of 100 k cells per well. Two to 3 days after seeding, the cells were fixed with 4% PFA (Nacalai-Tesque, Japan) for 15 min at room temperature (RT) and permeabilized with PBS containing 0.2% Triton X-100 (Promega, USA) for 15 min at RT. The cells were then exposed to blocking buffer consisting of 1% FBS for 1 h at RT. Next, cells were incubated with primary antibodies in blocking buffer for overnight at 4 °C, followed by incubation with appropriate fluorescence-tagged secondary antibodies at RT for 1 h in the dark. The primary antibodies used were rabbit-anti-human MLC2V (1:400; 10,906–1-AP; Proteintech) and mouse-anti-human MLC2A (1:200; 311 011; Synaptic Systems). The secondary antibodies used were Alexa Fluor 594-conjugated donkey anti-rabbit and Alexa Fluor 488-conjugated donkey anti-mouse (1:1000; Thermo Scientific). Nuclei were counterstained with 4′,6-diamidino-2-phenylindole (DAPI) (1:1000; AAT Biorequest, USA) at RT for 5 min. Cells were washed thrice in PBS between each step. Stained cells were visualized with Nikon ECLIPSE Ti-S fluorescent microscope and analysed with Nikon’s NIS-Elements AR analysis software.

### Western blot analysis

Cells were lysed in RIPA buffer (Thermo Scientific, USA) containing cOmplete™ Protease Inhibitor Cocktail (Sigma-Aldrich, USA). Protein concentration was quantitated with Pierce™ BCA Protein Assay Kit (Thermo Scientific, USA) in accordance to manufacturer’s recommendations. Protein lysates were resolved in 12% SDS-PAGE gels in Tris–Glycine–SDS buffer and transferred to a nitrocellulose membrane (Bio-Rad, USA) using the Trans-Blot® Turbo™ transfer system (Bio-Rad, USA). Membranes were blocked with 5% blotting-grade blocker (1,706,404; Bio-Rad) in tris-buffered saline with 0.1% tween20 (TBS-T) for 30 min and subsequently incubated with primary antibodies overnight at 4 °C. The following primary antibodies were used: rabbit-anti-human MLC2V (1:1000; 10,906–1-AP; Proteintech), mouse-anti-human MLC2A (1:1000; 311 011; Sypnatic Systems), rabbit-anti-GAPDH (1:1000; D16H11, Cell Signalling Technologies) and mouse-anti-GAPDH (1:1000; ab16048, Abcam). The blots were subsequently incubated with respective HRP-conjugated antibodies at 1:1000 dilution for 1 h at room temperature. Labelled proteins were detected with Clarity™ ECL Western Substrate (Bio-Rad, USA) and visualized with Fujifilm LAS-3000 imaging system (Fujifilm). Densitometry analysis was performed with Image Lab software (Bio-Rad, USA).

### Fluorescent imaging of calcium transient

Surface marker-sorted cardiomyocytes were seeded on a 96-well plate coated with Matrigel at a density of 80 k-100 k cells per well. Calcium transient imaging was performed after resumption of cell contraction was observed. Prior to imaging, the cells were washed twice with Tyrode’s Salt solution with 1 × sodium bicarbonate (Sigma-Aldrich, T2397). The cells were then incubated in Tyrode’s Salt solution containing 2.5 µM of the calcium indicator Fluo-4 AM (ThermoFisher, F14201) with 0.02% PluronicTM F-127 (ThermoFisher, P3000MP) for 45 min in a 5% CO2 incubator at 37◦C. Fluo-4 AM loading solution was then removed, the cells were washed and subsequently incubated in Tyrode’s Salt for another 20 min in the incubator before imaging. Calcium transients of sorted CMs were recorded using Nikon ECLIPSE Ti-S fluorescent microscope for 30 s and video data were analysed using Nikon’s NIS-Elements AR. Fluorescent intensity values were processed using our self-compiled code in R (RStudio version 1.2.1335/Base R version 3.6.3) to identify fluorescent peaks corresponding to single cardiac contraction [31].

### Vernakalant treatment

Surface marker-sorted cardiomyocytes seeded on 96-well plate (at density of 80–100 k cells per well) were incubated with the calcium indicator Fluo-4 AM loading solution as previously mentioned, and calcium transients imaging was recorded as data for pre-treatment samples. For post-treatment samples, after 45 min of incubation with the Fluo-4 AM loading solution, cells were then treated with 10 µM of vernakalant hydrochloride (HY-14183; MedChemExpress) in Tyrode’s Salt solution for 30 min, followed by calcium transients imaging. For withdrawal samples, vernakalant hydrochloride solution was removed by washing twice with Tyrode’s Salt solution before imaging.

### Statistical analysis

All the experimental data are presented as mean ± SD. Statistical differences of the data were determined using an unpaired Student’s t test. The equality of the variances was tested using an F test. All p values are two-tailed. A value of P < 0.05 was considered statistically significant.

## Results

### Potential surface markers shortlisted for purification of ventricular-specific cardiomyocytes

It is known that myosin regulatory light chain 2 encoded by MYL7 gene (also referred to as MLC2A) was predominantly expressed in all immature cardiomyocytes. In addition to MLC2A protein, immature CMs will gradually express MLC2V protein (encoded by MYL2 gene) as they mature towards ventricular fate, whereas atrial CMs will continue to express only MLC2A throughout the maturation process [32, 33]. As such, we reasoned that by conducting gene expression profiling on MLC2A- and MLC2V-expressing cardiomyocytes using RNA-seq approach, we could identify novel surface markers that aid in distinguishing ventricular cells from heterogeneous CMs culture.

**Fig. 1:**
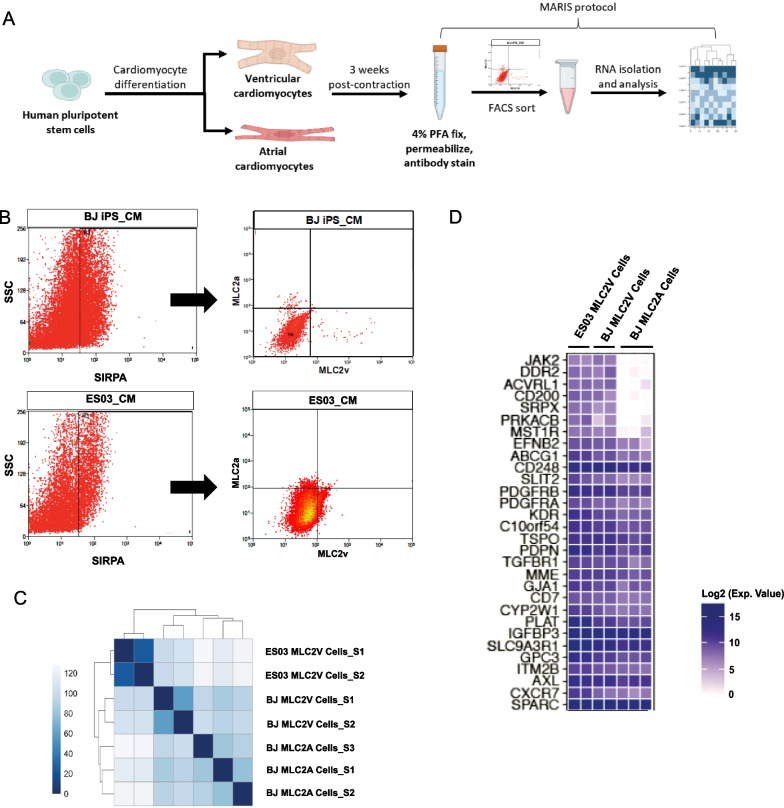
JAK2 and CD200 as potential surface markers for ventricular-specific cardiomyocytes. **A** Schematic diagram illustrating the protocol used to identify putative surface markers for ventricular-specific CMs. Created with BioRender.com. **B** Gating strategy for isolation of MLC2A + and MLC2V + cardiomyocytes derived from BJ and ESO3 lines by FACS. **C** Hierarchical clustering analysis of the top 500 differentially expressed genes revealed a distinct separation between MLC2V + and MLC2A + samples. **D** Heatmap showing the list of putative surface marker genes for ventricular-specific cardiomyocytes identified through RNA sequencing analysis

As shown in Fig. 1A, cardiomyocytes were first differentiated from 2 hPSC lines (BJ and ES03) using published protocol [17]. Induction of cardiac differentiation using this method resulted in beating cardiomyocytes at Day 9 of culture. Three weeks post initial contraction, cardiomyocytes were labelled with SIRPA, intracellular MLC2A and MLC2V antibodies using MARIS [19, 20], followed by FACS to isolate MLC2A + and MLC2V + cardiomyocytes. Figure 1B showed the FACS plots of the gating strategy for isolation of MLC2A + and MLC2V + cardiomyocytes. RNA sequencing was then conducted to examine the gene expression profile of MLC2A + and MLC2V + cardiomyocytes. The results revealed a significant difference in the gene expression between the two subtypes of CMs derived from BJ line, with a total of 2865 genes showing differential expression. Of these, 1349 and 1516 genes were significantly up- and down-regulated, respectively, in the BJ MLC2V + samples compared to BJ MLC2A + samples (Additional file 1: Table S1). Additionally, a comparison of differentially expressed genes was also performed between ESO3 MLC2V + and BJ MLC2A + samples, revealing that 1904 and 2811 genes were significantly up- and down-regulated, respectively (Additional file 2: Table S2). The top 500 genes across the samples were used to conduct hierarchical clustering, and the resulting dendrogram (Fig. 1C) clearly demonstrated a distinct separation between MLC2V-sorted CMs samples and MLC2A-sorted CMs samples, regardless of the PSC lines that were used.

To identify potential surface marker candidates that play a role in ventricular specification, we focused on genes that were significantly up-regulated in MLC2V + samples, as compared to MLC2A + samples. To further refine the list of potential surface marker candidates, only genes that were up-regulated in both BJ and ES03 MLC2V + samples were taken into consideration. By cross-referencing these commonly up-regulated genes in both BJ and ES03 MLC2V + samples with our previous research findings on the list of membrane receptors expressed in hESCs [34] and GeneCards human gene database (http://www.genecards.org), we discovered 30 putative surface markers for ventricular-specific cardiomyocytes (Additional file 3: Table S3), as presented in Fig. 1D. Among these candidates, JAK2 and CD200 were selected for further investigation.

### SIRPA+/JAK2 + and SIRPA+/CD200 + populations were enriched for ventricular-like cardiomyocytes

To assess the effectiveness of the putative cell surface markers in purifying ventricular-specific cardiomyocytes, FACS sorting was employed to isolate different subsets of cardiomyocytes expressing SIRPA alone, SIRPA+/JAK2+, SIRPA+/JAK2−, SIRPA+/CD200+ and SIRPA+/CD200−. Analysis via immunofluorescent staining of SIRPA + only cardiomyocytes indicated the presence of a mixed population of MLC2V- and MLC2A-expressing cells (Additional file 4: Fig. S1). Specifically, for BJ-derived CMs, approximately 48 ± 12% and 52 ± 10% of SIRPA + CMs expressed MLC2V and MLC2A, respectively. Similar findings were obtained for H7-derived CMs, where 43 ± 6% and 57 ± 6% of SIRPA + cells were MLC2V + and MLC2A + , respectively. These results are consistent with previous reports where sorting with SIRPA alone did not result in specific cardiac subtype enrichment [35].

**Fig. 2:**
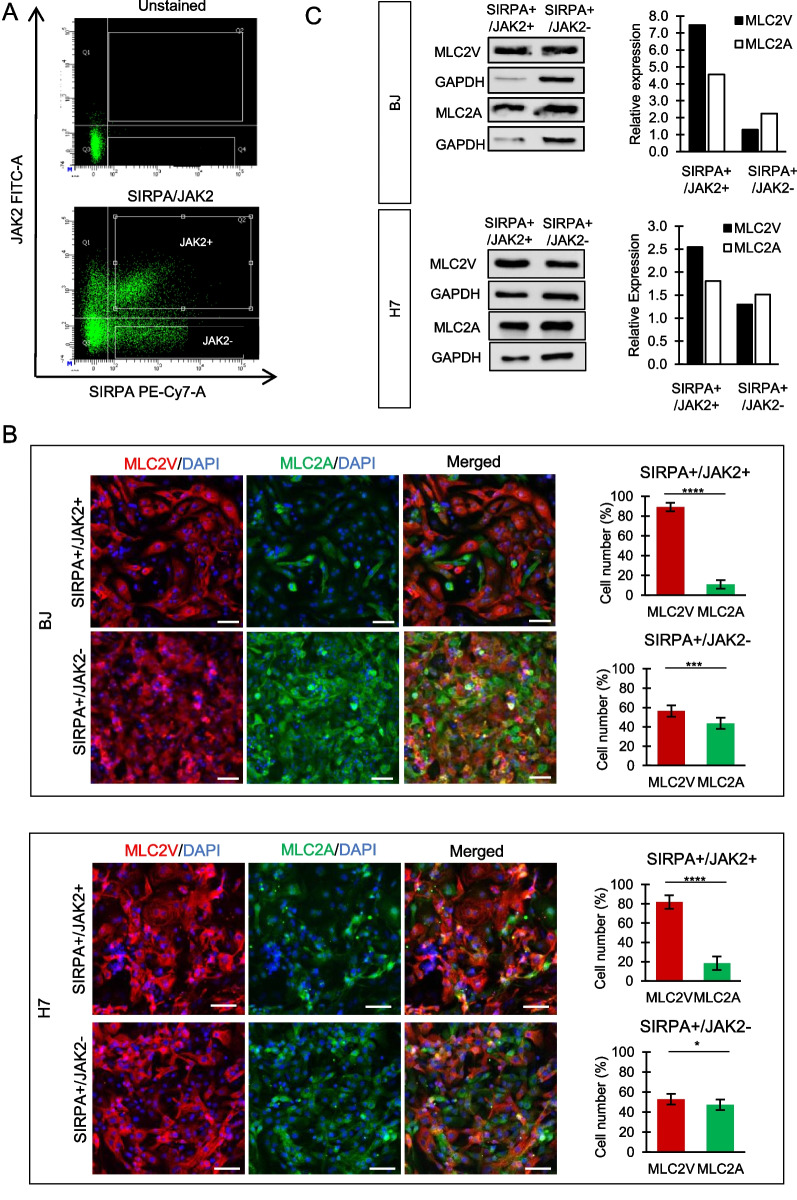
SIRPA+/JAK2 + population shows enrichment of ventricular CMs. **A** Representative FACS plots of CMs sorted using SIRPA and JAK2 antibodies. Unstained CMs served as negative control. **B** Representative images of SIRPA+/JAK2 + (top panel) and SIRPA+/JAK2− (bottom panel) CMs co-stained with MLC2V and MLC2A antibodies. Nucleus was stained with DAPI. The percentage of MLC2V + and MLC2A + CMs in the respective populations was quantified and expressed as mean ± SD. *P < 0.05, **P < 0.01, ***P < 0.001, ****P < 0.0001 (t test). Data were collected from duplicate experiments. Scale bar = 100 µm. **C** Western blot analysis of MLC2A and MLC2V expression in SIRPA+/JAK2 + and SIRPA+/JAK2− populations, normalized to GAPDH expression. Full-length blots are presented in Additional file 5: Fig. S2 (for BJ SIRPA/JAK2 sorted samples) and Additional file 6: Fig. S3 (for H7 SIRPA/JAK2 sorted samples)

**Fig. 3:**
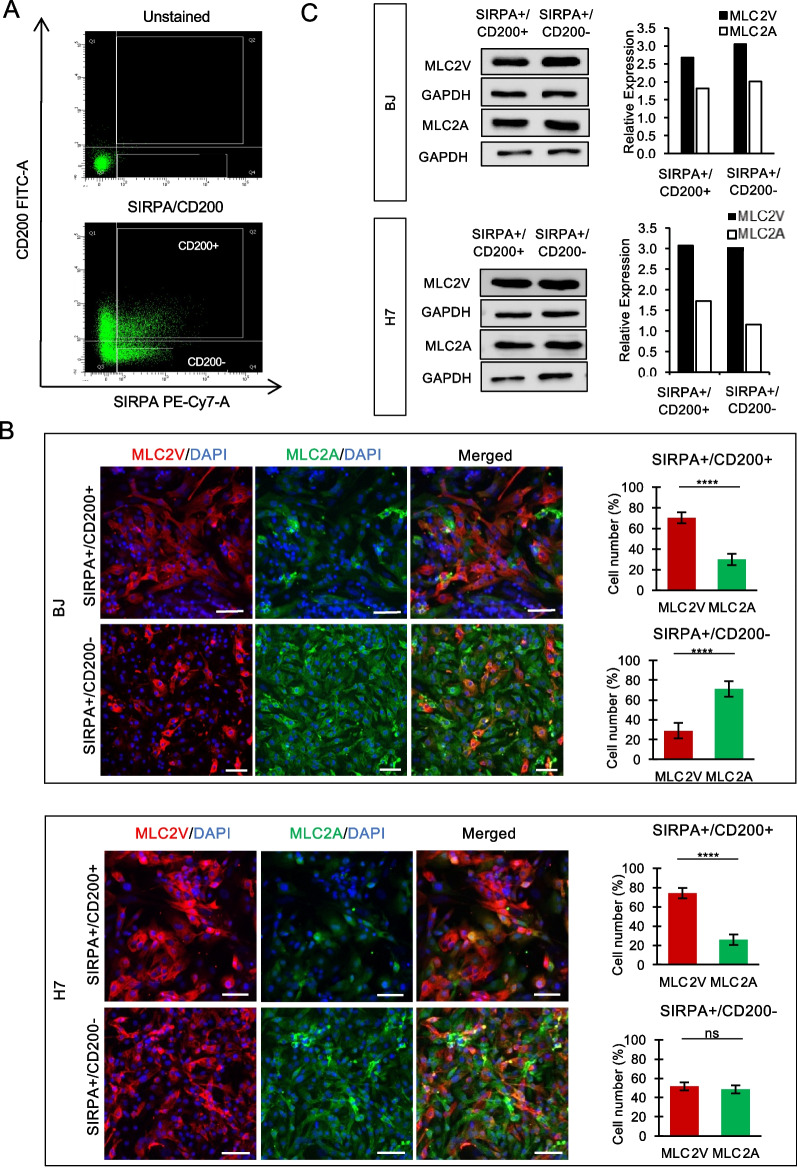
SIRPA+/CD200 + population shows enrichment of ventricular CMs. **A** Representative FACS plots of CMs sorted using SIRPA and CD200 antibodies. Unstained CMs served as negative control. **B** Representative images of SIRPA+/CD200 + (top panel) and SIRPA+/CD200− (bottom panel) CMs co-stained with MLC2V and MLC2A antibodies. Nucleus were stained with DAPI The percentage of MLC2V + and MLC2A + CMs in the respective populations was quantified and expressed as mean ± SD. *P < 0.05, **P < 0.01, ***P < 0.001, ****P < 0.0001 (t test). Data were collected from duplicate experiments. Scale bar = 100 µm. **C** Western blot analysis of MLC2A and MLC2V expression in SIRPA+/CD200 + and SIRPA+/CD200− populations, normalized to GAPDH expression. Full-length blots are presented in Additional file 6: Fig. S3 (for BJ SIRPA/CD200 sorted samples) and Additional file 7: Fig. S4 (for H7 SIRPA/CD200 sorted samples)

On the contrary, we demonstrated that purification based on SIRPA/JAK2 expression led to an enrichment of ventricular-like CMs, as evidenced by a higher percentage of MLC2V-expressing cells (89 ± 4% and 82 ± 7%) in the SIRPA+/JAK2+ cell population compared to the SIRPA+/JAK2− population (56 ± 5% and 53 ± 5% of MLC2V + cells) in both BJ and H7 cell lines, respectively (Fig. 2B). Similarly, isolation of SIRPA+/CD200 + CMs resulted in up to 70 ± 5% (BJ) and 74 ± 5% (H7) purity of ventricular-like CMs (Fig. 3B).

Consistent findings were observed between western blot results and immunofluorescent staining. We noted an increased expression of the ventricular-specific MLC2V protein and decreased expression of MLC2A proteins in the SIRPA+/JAK2+ (Fig. 2C, Additional file 5: Fig. S2 and Additional file 6: Fig. S3) or SIRPA+/CD200+ (Fig. 3C, Additional file 6: Fig. S3 and Additional file 7: Fig. S4) sorted population in both cell lines.

### SIRPA +/JAK2 + and SIRPA +/CD200 + sorted cardiomyocytes displayed electrophysiological properties similar to the ventricular-like cardiomyocytes

**Fig. 4:**
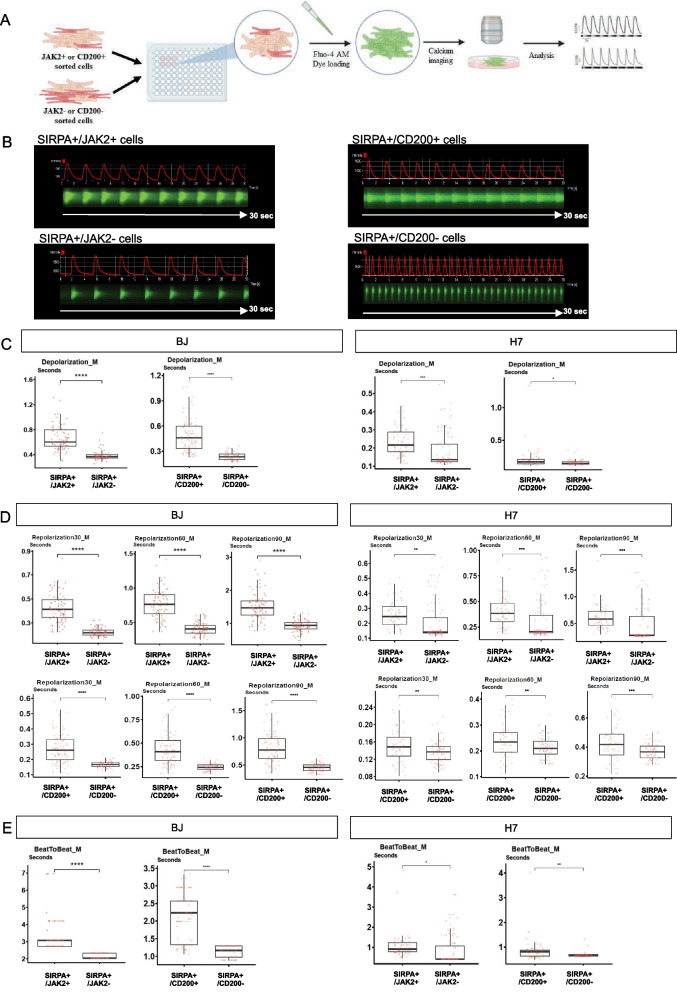
Electrophysiological characterization of SIRPA + CMs sorted with JAK2 or CD200 surface markers. **A** Workflow of the calcium imaging analysis for surface markers-sorted CMs. Created with BioRender.com. **B** Representative kymographs and the corresponding calcium transient profiles for CMs sorted with JAK2 and CD200 surface markers. **C, D, E** Dot plot showing mean of **C** calcium depolarization duration, **D** calcium repolarization duration at 30%, 60% and 90%, and **E** beat-to-beat duration in SIRPA/JAK2 and SIRPA/CD200 sorted cardiomyocytes. *P < 0.05, **P < 0.01, ***P < 0.001, ****P < 0.0001 (t test)

Atrial and ventricular cardiomyocytes exhibit distinct calcium transient profiles [36]. To characterize the electrophysiological properties of surface markers-sorted cardiomyocytes, we utilized fluo-4 AM, a green-fluorescent calcium indicator, to measure the cellular calcium transient at the single cell level. We analysed various parameters, including beat-to-beat duration, depolarization and repolarization phase duration (Fig. 4A and Additional file 8: Fig. S5). Through calcium imaging analysis, we observed distinct calcium transient morphology in both the positive- and negative-sorted populations, regardless of the surface marker used (Fig. 4B). Notably, the majority of CMs sorted positive for JAK2 and CD200 surface markers displayed a significantly slower depolarization time (time to peak) compared to SIRPA+/JAK2− and SIRPA+/CD200− populations (Fig. 4C). Additionally, SIRPA+/JAK2+ and SIRPA+/CD200+ CMs exhibited a significantly longer repolarization duration at 30%, 60% and 90%, indicating ventricular-like electrophysiological properties (Fig. 4D). Conversely, SIRPA+/JAK2− and SIRPA+/CD200− populations contained a considerable number of CMs with a significantly shorter repolarization time, indicative of human atrial-like electrophysiology. Furthermore, when comparing beat-to-beat durations, we observed pronounced differences in beating frequency. Specifically, SIRPA+/JAK2+ and SIRPA+/CD200+ populations exhibited slower beating rates compared to their respective negative populations, resembling the properties of CMs from the human ventricle (Fig. 4E).

### SIRPA+/JAK2+ and SIRPA+/CD200+ CMs did not respond to vernakalant treatment, an atrial-selective anti-arrhythmic agent

Finally, we performed functional validation on the surface markers-sorted cardiomyocytes by investigating their response towards treatment with vernakalant. Vernakalant is an anti-arrhythmic agent that selectively targets atrial CMs by blocking the potassium channels and prolonging the repolarization phase in these cells [37]. Given this mechanism of action, it is therefore reasonable to infer that vernakalant treatment would primarily affect the population containing a significant amount of atrial CMs, rather than ventricular CMs.

**Fig. 5:**
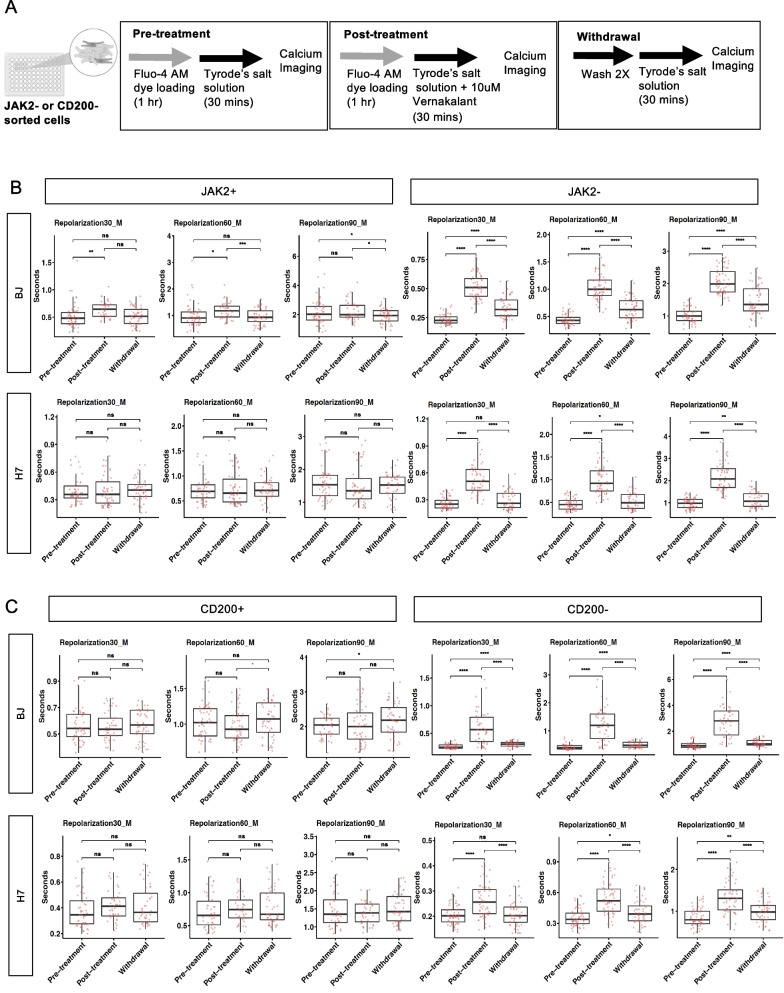
Electrophysiological characterization of SIRPA + CMs sorted with JAK2 or CD200 surface markers treated with vernakalant. **A** Schematic showing calcium imaging workflow for the evaluation of the effects of vernakalant on CMs sorted with surface markers. Created with BioRender.com. **B, C** Effect of 10 µm vernakalant treatment on the mean of repolarization duration (at 30%, 60% and 90%) in **B** SIRPA/JAK2 and **C** SIRPA/CD200 sorted cardiomyocytes. *P < 0.05, **P < 0.01, ***P < 0.001, ****P < 0.0001 (t test)

In this study, we treated the surface marker-sorted CMs with 10 µM vernakalant and assessed their calcium transient profile at three different stages, namely the pre-treatment, post-treatment and withdrawal stages of vernakalant (see Fig. 5A) [38]. We observed a distinct change in the calcium transient profile of the CMs from the SIRPA+/JAK2− and SIRPA+/CD200− populations upon treatment with vernakalant. Specifically, the repolarization duration of these CMs at 30%, 60% and 90% was significantly prolonged in both BJ and H7 cell lines (right panels in Fig. 5B and 5C). This effect was reversed upon removal of vernakalant through a repeated washing procedure. In contrast, CMs from the SIRPA+/JAK2+ and SIRPA+/CD200+ populations exhibited minimal to no significant response to vernakalant, indicating an enrichment of ventricular-like CMs in these populations (left panels in Fig. 5B, C).

Although we observed a slight increase in the repolarization duration at 30% and 60% in BJ SIRPA+/JAK2+ cardiomyocytes following vernakalant treatment, we considered this effect to be negligible as the extent of elongation was very minor when compared to the significant changes observed in BJ SIRPA+/JAK2− cells.

## Discussion

The generation and purification of cardiomyocyte sub-population are critical for precise modelling of cardiac diseases, drug screening and regenerative medicine development due to their distinct phenotypic and functional characteristics. Therefore, identifying surface markers for cardiomyocyte subtype that facilitate live cell sorting of homogenous population of cardiomyocytes is of utmost importance for successful translation of cardiovascular research and therapeutic applications.

In the present study, we identified CD200 and JAK2 as potential candidate surface markers for purifying ventricular CMs. CD200 is a cell surface glycoprotein belonging to the immunoglobulin superfamily [39], while JAK2 is a member of the Janus kinase (JAKs) family with several reports suggested different localization, including cytoplasm [40], cell surface or plasma membrane [41, 42]. While our study reported CD200 to be a potential cell surface marker for ventricular CM, an earlier study by Veevers et al. reported that CD200 is negatively associated with ventricular CMs [43]. In that study, the authors used a panel of 242 known anti-human monoclonal antibodies to screen through a hPSC line harbouring GFP reporter driven by MYL2 promoter to show that CD77+/CD200− are cell surface markers for ventricular CMs. While they showed that 65% of the CD77+/CD200− population expressed MYL2-GFP, suggesting that they are ventricular CMs, it was subsequently reported that there is little to no expression of CD77 detected in CMs derived from two independent wildtype iPSC lines, hampering its application in all hPSC lines [43].

In the current study, we have employed a different approach that utilized MARIS, coupled with RNA sequencing analysis on MLC2A- and MLC2V-expressing cardiomyocytes to identify potential surface markers that can be used to isolate specific cardiomyocyte sub-populations. This method enables profiling of rare cell types that are difficult to isolate using traditional sorting approaches while maintaining cellular integrity and improving the accuracy of gene expression analysis. Importantly, isolation of CMs through the use of cell surface markers eliminates the need for genetic modification, which in turn minimizes safety concerns such as the inadvertent activation of oncogenes or the introduction of novel allergenic or immunogenic elements. This is particularly relevant for therapeutic applications such as cell transplantation, where safety is of upmost importance.

While we identified CD200 as one of the potential ventricular cell surface markers, we achieved a marginal increase in the enrichment of ventricular-like CMs (70%-75%) when sorting together with SIRPA antibody (SIRPA+/CD200+) as compared to the unsorted (SIRPA + only) population (up to 43%-48%). In contrast, purification based on SIRPA+/JAK2+ expression led to a significant enrichment of ventricular-like CMs (~ 90%). To further validate these surface markers, we utilized MYL2-TdTomato reporter system generated from H7 embryonic stem cell (ESC) line (H7-MYL2-TdTomato). Remarkably, among the TdTomato-positive cardiomyocytes, 71.25% exhibited co-expression of SIRPA and JAK2, while 48.5% displayed co-expression of SIRPA and CD200 (Additional file 9: Fig. S6). This additional finding further supports our assertion regarding the potential of SIRPA/JAK2 cell surface markers in facilitating the isolation of ventricular cardiomyocytes. Interestingly, while CD200 and JAK2 expressions are not mutually exclusive in the ventricular cardiomyocyte sub-population, the utility of using both CD200 and JAK2 did not produce a synergistic effect in enhancing the purity of ventricular cardiomyocytes (data not shown). At the molecular level, RNA sequencing revealed that gene expression profiles of SIRPA+/JAK2+ cardiomyocytes closely resembled those of H7-MYL2-TdTomato + cardiomyocytes. In addition, SIRPA+/JAK2+ CMs exhibited high expression levels of ventricular-associated genes previously reported [44–46], including MYL2, HEY2, IRX4 and DLK1, comparable to ventricular CMs generated from reporter line and adult human ventricle samples (Additional file 10: Fig. S7).

## Conclusion

Taken together, our study has identified CD200 and JAK2 as potential surface markers for ventricular-like cardiomyocytes, with SIRPA+/JAK2+ achieving a higher purity in isolating ventricular subtype. The results illustrated JAK2 as a novel surface marker for the purification of stem cells-derived ventricular CM. By enhancing the purity of ventricular CMs, it could potentially improve drug screening processes or transplantation outcome by mitigating the risk of arrhythmic phenotype.

### Supplementary Information

**Additional file 1: Table S1.** List of differentially expressed genes between BJ MLC2V + and BJ MLC2A + cardiomyocytes identified by RNA-Seq analysis.

**Additional file 2: Table S2.** List of differentially expressed genes between ES03 MLC2V + and BJ MLC2A + cardiomyocytes identified by RNA-Seq analysis.

**Additional file 3: Table S3.** List of putative surface markers for isolation of ventricular-specific cardiomyocytes.

**Additional file 4: Figure S1.** Representative images of SIRPA + only CMs co-stained with MLC2V and MLC2A antibodies, followed by quantification of the percentage of MLC2V + and MLC2A + CMs. All values are expressed as mean ± SD. *P < 0.05, **P < 0.01, ***P < 0.001, ****P < 0.0001 (t test). Data were collected from duplicate experiments.

**Additional file 5: Figure S2.** Full-length blots of MLC2V (left blot), MLC2A (right blot) and the respective GAPDH expressions in SIRPA+/JAK2+ and SIRPA+/JAK2− populations for BJ cell line. Red boxes indicate the cropped blots shown in Fig. 2C.

**Additional file 6: Figure S3.** Full-length blots of MLC2V (left blot), MLC2A (right blot) and the respective GAPDH expressions in SIRPA+/JAK2+, SIRPA+/JAK2−, SIRPA+/CD200+ and SIRPA+/CD200− populations. Red boxes indicate the cropped blots shown in Fig. 2C (H7 SIRPA/JAK2) and Fig. 3C (BJ SIRPA/CD200).

**Additional file 7: Figure S4.** Full-length blots of MLC2V (left blot), MLC2A (right blot) and the respective GAPDH expressions in SIRPA+/CD200+ and SIRPA+/CD200− populations for H7 cell line. Red boxes indicate the cropped blots shown in Fig. 3C.

**Additional file 8: Figure S5.** Schematic showing calcium transient profile derived per single cardiomyocyte contraction rhythm. The diagram illustrates the various parameters that were measured in each cardiomyocyte contraction cycle, including calcium transient amplitude, depolarisation and repolarization phase duration.

**Additional file 9: Figure S6**. Validation of surface markers-sorted CMs using MYL2 reporter line. **A** Representative FACS plots showing the co-staining of SIRPA/JAK2 or SIRPA/CD200 antibodies in H7-MYL2-TdTomato-derived CMs. Unstained CMs derived from H7 wild type were used as a negative control. **B** Quantification of the percentage of SIRPA+/JAK2+ and SIRPA+/CD200+ cells within the TdTomato + population, presented as mean ± SD. The data were obtained from duplicate experiments.

**Additional file 10: Figure S7.** RNA sequencing analysis comparing gene expression profiles across different samples. **A** Heatmap illustrating the top 100 of atrial and ventricular genes in human adult atria and ventricle tissue. Heatmap depicting the expression of top 100 ventricular genes in SIRPA+/JAK2+ cardiomyocytes, in comparison with **B** H7-MYL2-TdTomato + cardiomyocytes and **C** samples obtained from the adult human atria and ventricle of the heart. **D** Assessment of ventricular- and atrial-specific gene expressions across diverse sample sets.

## Data Availability

The data discussed in this publication have been deposited in NCBI's Gene Expression Omnibus and are accessible through GEO Series accession number GSE231913 (https://www.ncbi.nlm.nih.gov/geo/query/acc.cgi?acc=GSE231913).

## Abbreviations

hPSC: Human pluripotent stem cell
CMs: Cardiomyocytes
MLC2A: Myosin light chain 2A
MLC2V: Myosin light chain 2 V
JAK2: Janus kinase 2
hESCs: Human embryonic stem cells
hiPSCs: Human-induced pluripotent stem cells
CVDs: Cardiovascular diseases
COUP-TFII: Chick ovalbumin upstream promoter transcription factor II
MARIS: Method for analysing RNA following intracellular sorting
GFAM: Glucose-depleted culture medium supplemented with fatty acid
GSK-3: Glycogen synthase kinase 3
BSA: Bovine serum albumin
PBS: Phosphate-buffered saline
DPBS: Dulbecco’s phosphate-buffered saline
FACS: Florescence activated cell sorting
FBS: Foetal bovine serum
RT: Room temperature
SD: Standard deviation
SIRPA: Signal regulatory protein alpha
