# Supplementary figures and images for: JAK2 as a surface marker for enrichment of human pluripotent stem cells-derived ventricular cardiomyocytes
Source: Stem Cell Res Ther. 2023 Dec 13;14:367. doi: 10.1186/s13287-023-03610-2 (PMC10720068; doi:10.1186/s13287-023-03610-2)

Additional file 4: Supplementary figure 1

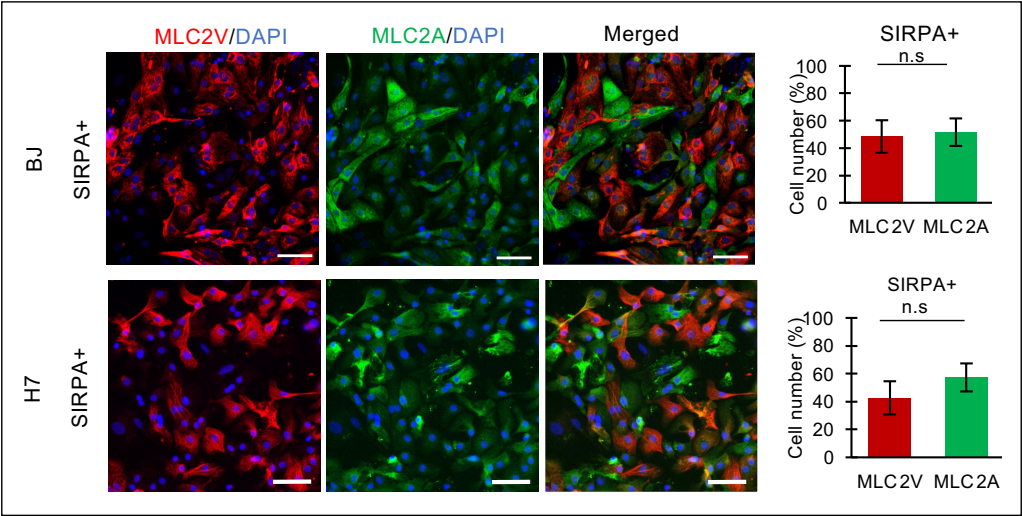

Supplement: Supplementary file 4 — Additional file 4: Figure S1. Representative images of SIRPA + only CMs co-stained with MLC2V and MLC2A antibodies, followed by quantification of the percentage of MLC2V + and MLC2A + CMs. All values are expressed as mean ± SD. *P < 0.05, **P < 0.01, ***P < 0.001, ****P < 0.0001 (t test). Data were collected from duplicate experiments. [file 13287_2023_3610_MOESM4_ESM.pdf]

Additional file 5: Supplementary figure 2

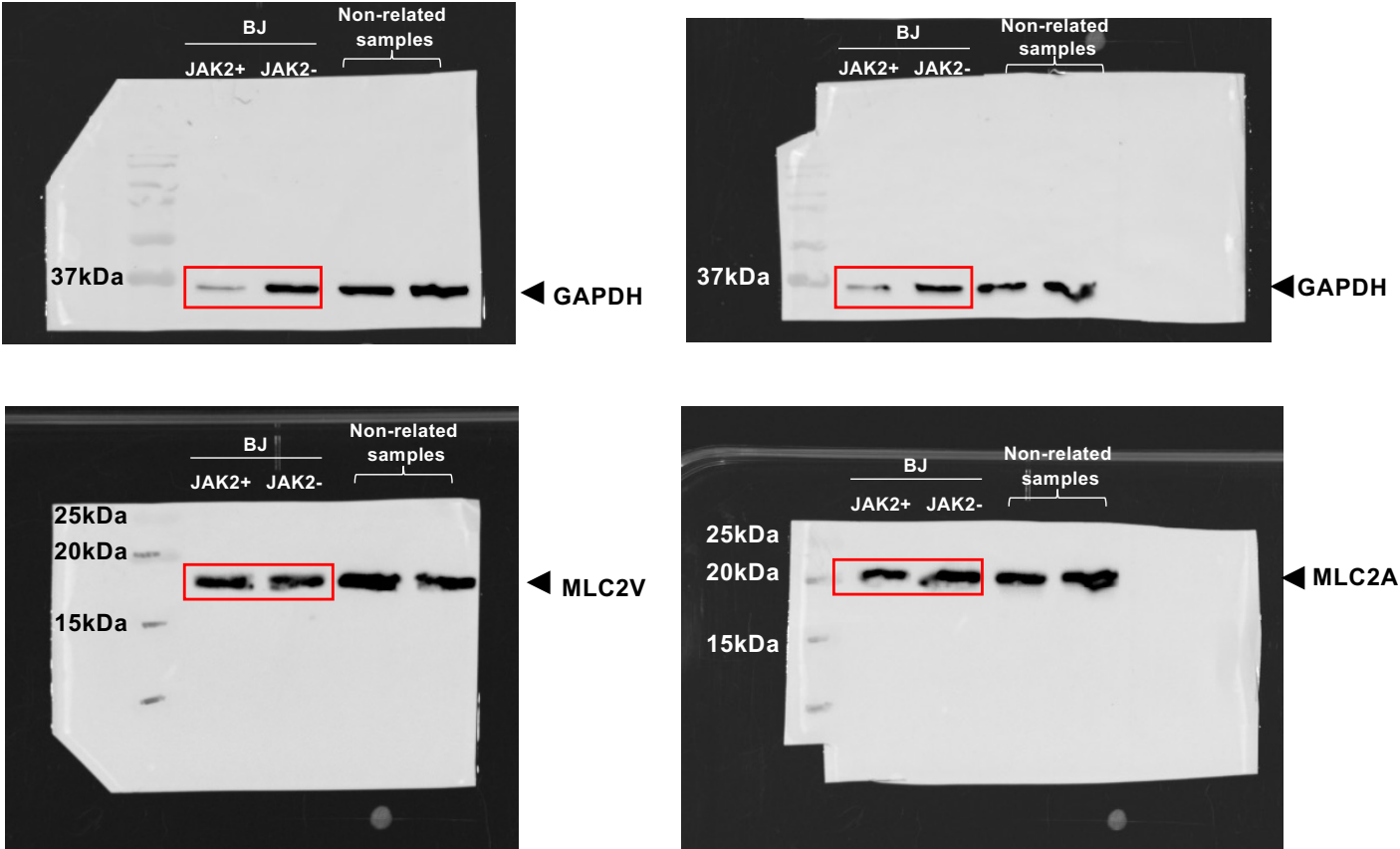

Supplement: Supplementary file 5 — Additional file 5: Figure S2. Full-length blots of MLC2V (left blot), MLC2A (right blot) and the respective GAPDH expressions in SIRPA+/JAK2+ and SIRPA+/JAK2− populations for BJ cell line. Red boxes indicate the cropped blots shown in Fig. 2C. [file 13287_2023_3610_MOESM5_ESM.pdf]

Additional file 6: Supplementary figure 3

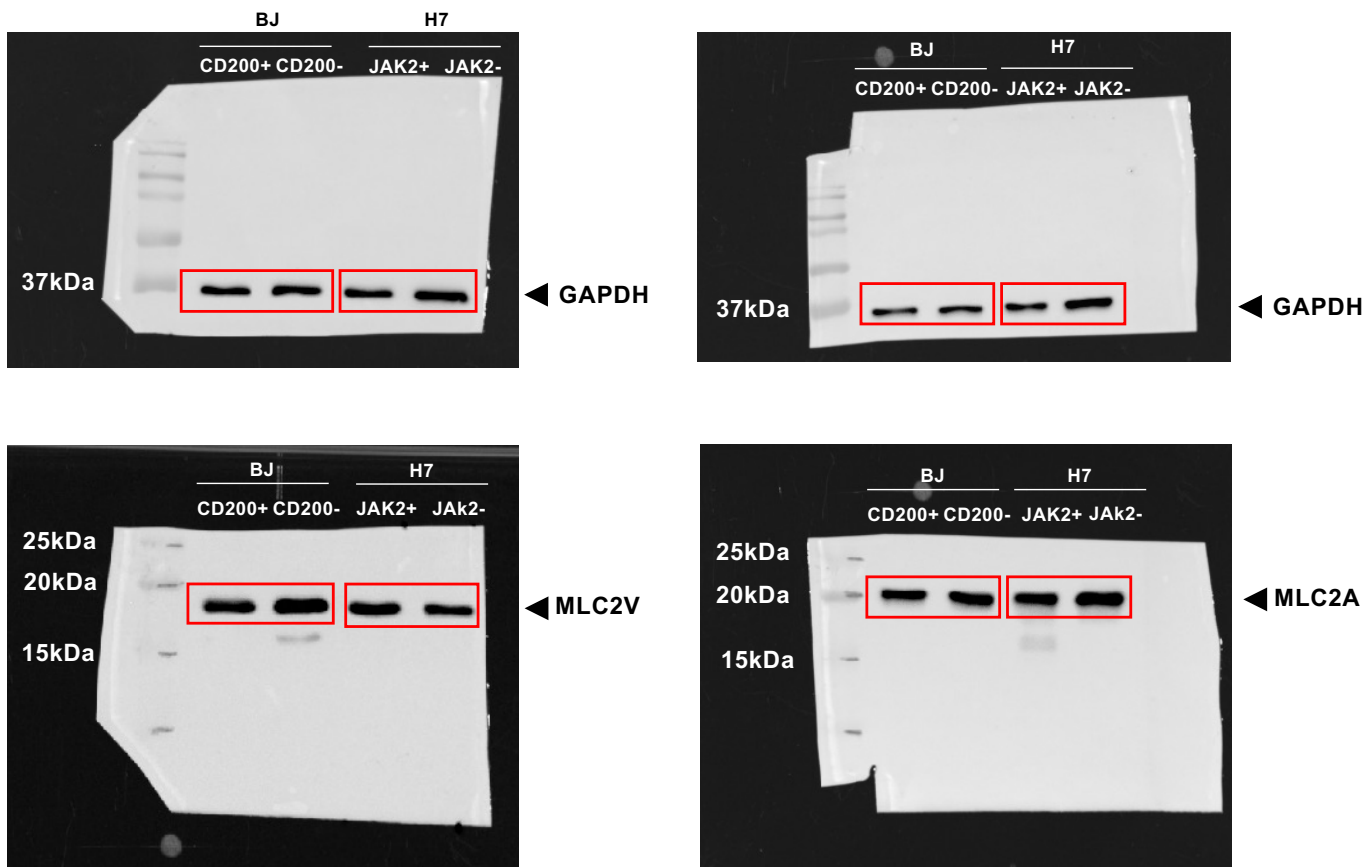

Supplement: Supplementary file 6 — Additional file 6: Figure S3. Full-length blots of MLC2V (left blot), MLC2A (right blot) and the respective GAPDH expressions in SIRPA+/JAK2+, SIRPA+/JAK2−, SIRPA+/CD200+ and SIRPA+/CD200− populations. Red boxes indicate the cropped blots shown in Fig. 2C (H7 SIRPA/JAK2) and Fig. 3C (BJ SIRPA/CD200). [file 13287_2023_3610_MOESM6_ESM.pdf]

Additional file 7: Supplementary figure 4

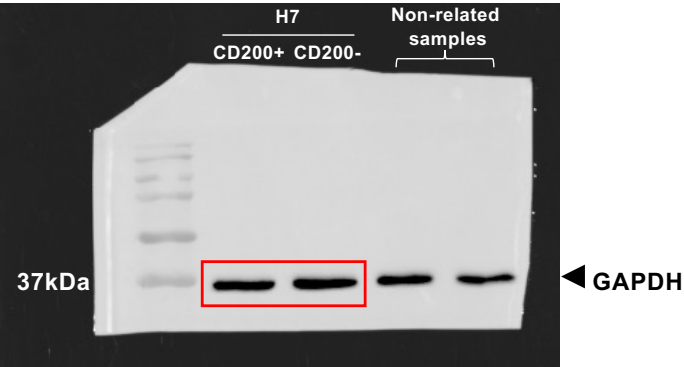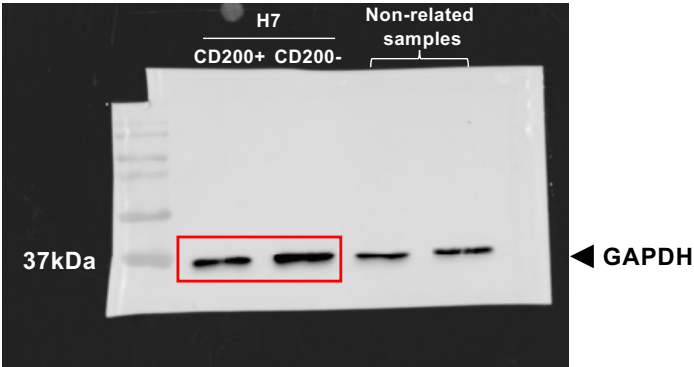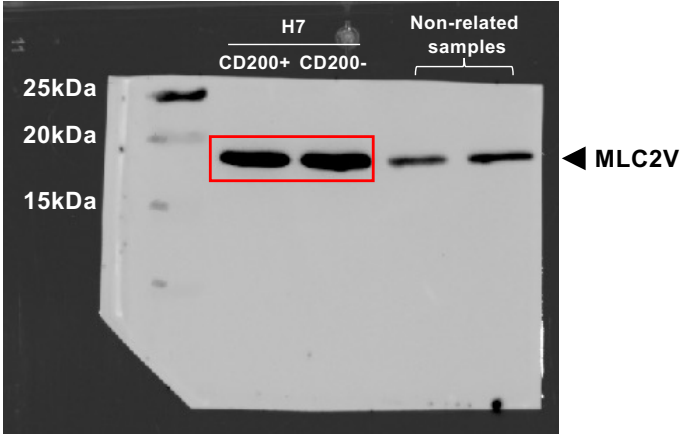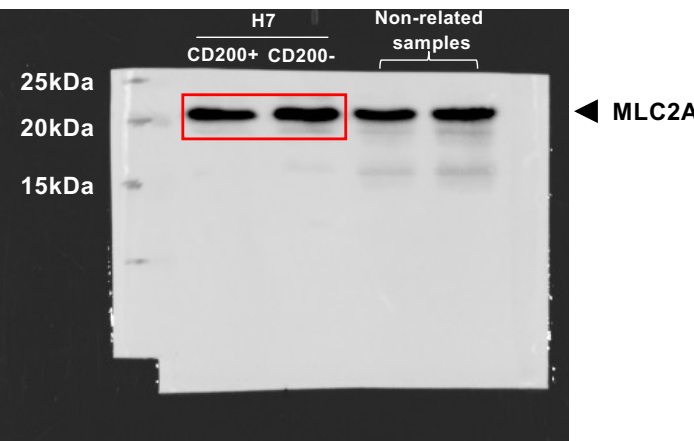

Supplement: Supplementary file 7 — Additional file 7: Figure S4. Full-length blots of MLC2V (left blot), MLC2A (right blot) and the respective GAPDH expressions in SIRPA+/CD200+ and SIRPA+/CD200− populations for H7 cell line. Red boxes indicate the cropped blots shown in Fig. 3C. [file 13287_2023_3610_MOESM7_ESM.pdf]

Additional file 8: Supplementary figure 5

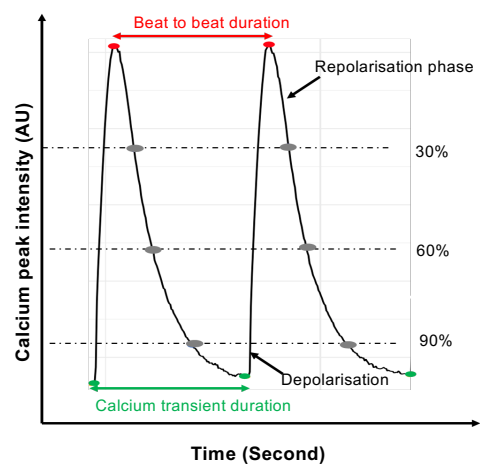

Supplement: Supplementary file 8 — Additional file 8: Figure S5. Schematic showing calcium transient profile derived per single cardiomyocyte contraction rhythm. The diagram illustrates the various parameters that were measured in each cardiomyocyte contraction cycle, including calcium transient amplitude, depolarisation and repolarization phase duration. [file 13287_2023_3610_MOESM8_ESM.pdf]

Additional file 9: Supplementary figure 6

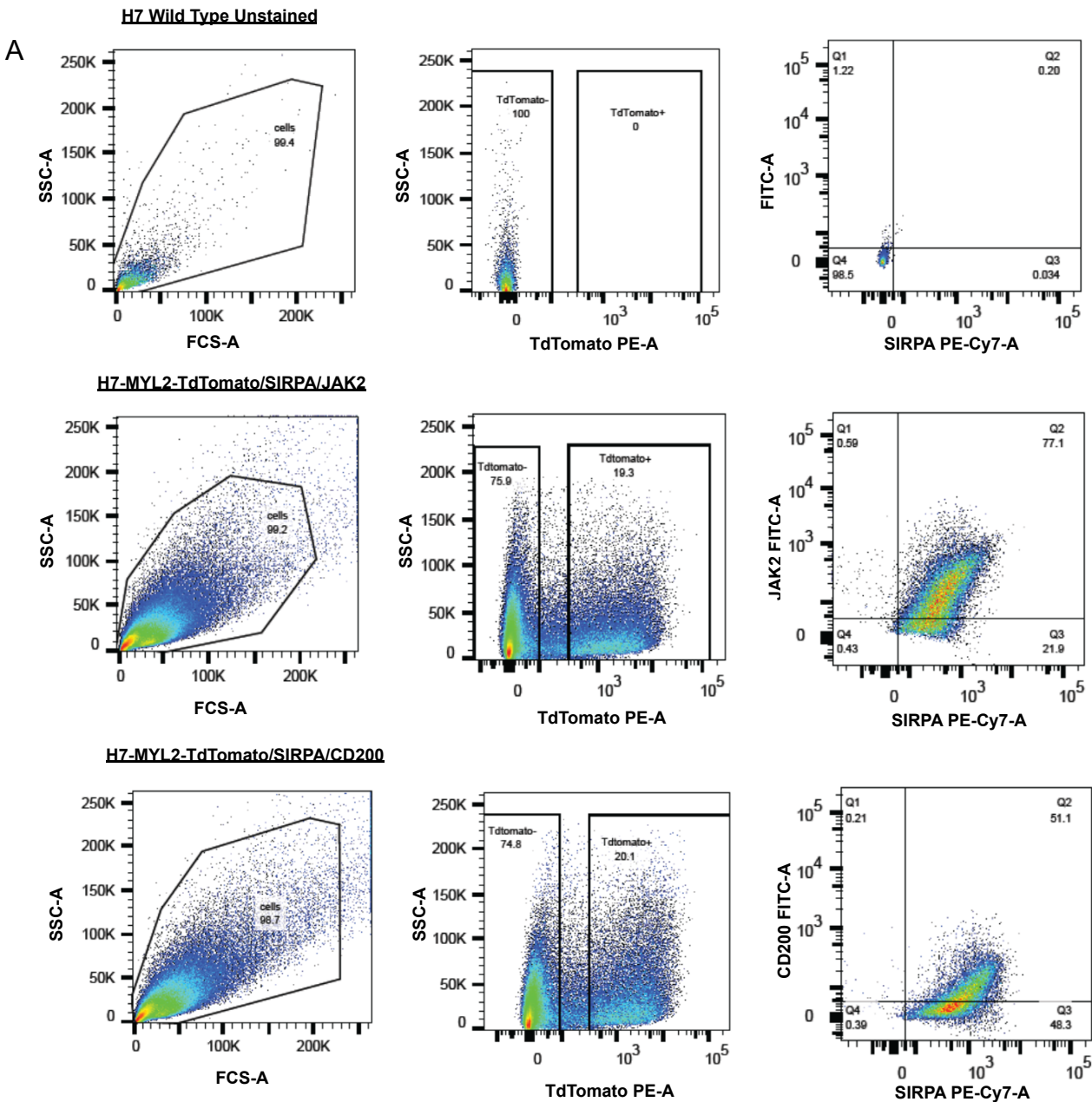

Supplement: Supplementary file 9 — Additional file 9: Figure S6. Validation of surface markers-sorted CMs using MYL2 reporter line. A Representative FACS plots showing the co-staining of SIRPA/JAK2 or SIRPA/CD200 antibodies in H7-MYL2-TdTomato-derived CMs. Unstained CMs derived from H7 wild type were used as a negative control. B Quantification of the percentage of SIRPA+/JAK2+ and SIRPA+/CD200+ cells within the TdTomato + population, presented as mean ± SD. The data were obtained from duplicate experiments. [file 13287_2023_3610_MOESM9_ESM.pdf]

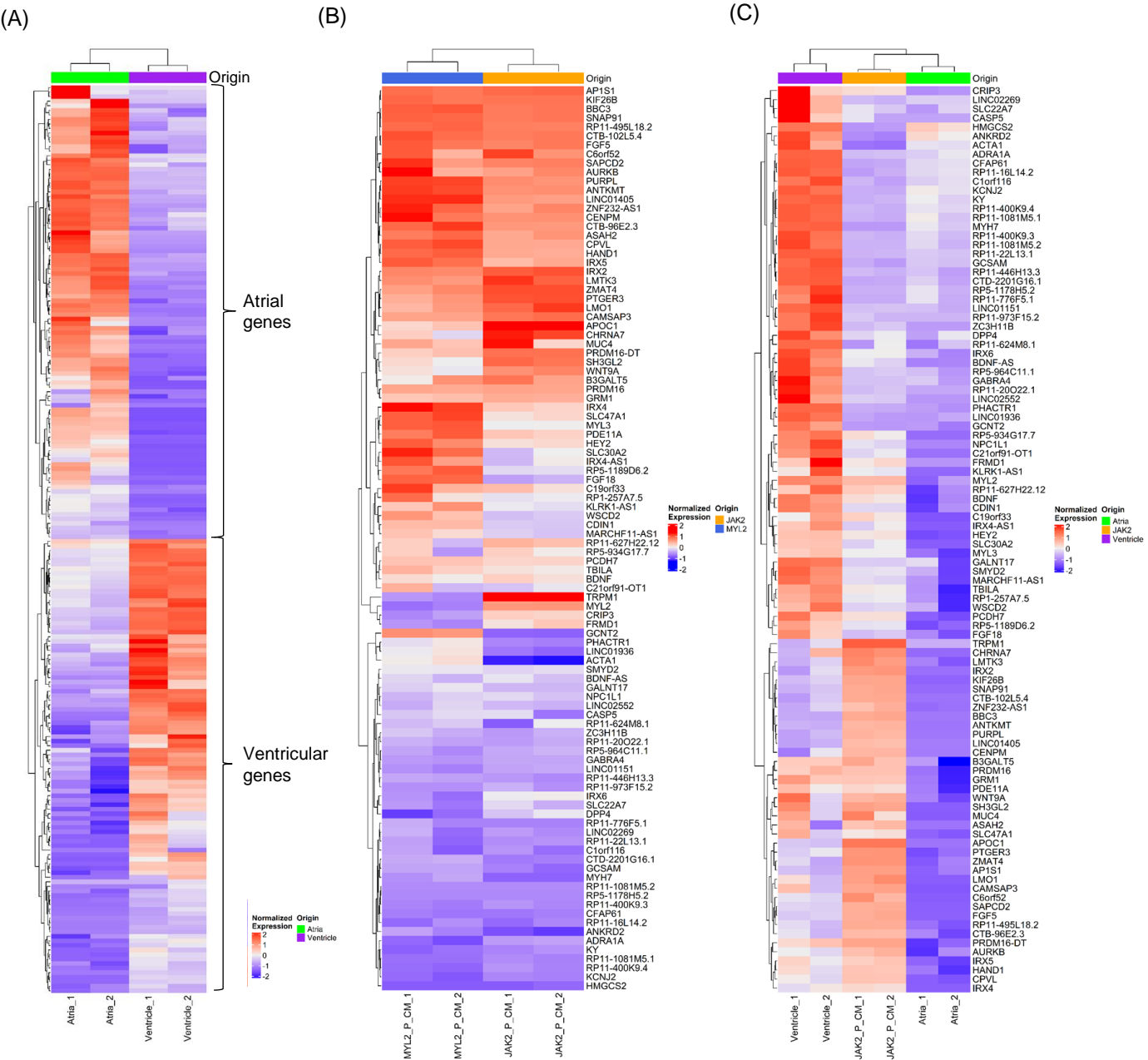

(D)

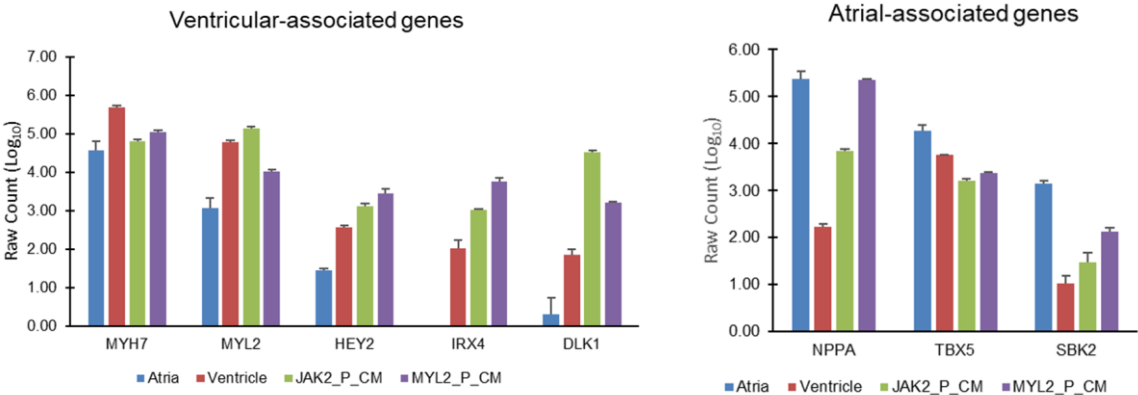

Supplement: Supplementary file 10 — Additional file 10: Figure S7. RNA sequencing analysis comparing gene expression profiles across different samples. A Heatmap illustrating the top 100 of atrial and ventricular genes in human adult atria and ventricle tissue. Heatmap depicting the expression of top 100 ventricular genes in SIRPA+/JAK2+ cardiomyocytes, in comparison with B H7-MYL2-TdTomato + cardiomyocytes and C samples obtained from the adult human atria and ventricle of the heart. D Assessment of ventricular- and atrial-specific gene expressions across diverse sample sets. [file 13287_2023_3610_MOESM10_ESM.pdf]
